# Phenotyping of *Arabidopsis* Drought Stress Response Using Kinetic Chlorophyll Fluorescence and Multicolor Fluorescence Imaging

**DOI:** 10.3389/fpls.2018.00603

**Published:** 2018-05-11

**Authors:** Jieni Yao, Dawei Sun, Haiyan Cen, Haixia Xu, Haiyong Weng, Fang Yuan, Yong He

**Affiliations:** ^1^College of Biosystems Engineering and Food Science, Zhejiang University, Hangzhou, China; ^2^Key Laboratory of Spectroscopy Sensing, Ministry of Agriculture, Hangzhou, China; ^3^Center for Plant Environmental Sensing, College of Life and Environmental Sciences, Hangzhou Normal University, Hangzhou, China

**Keywords:** kinetic chlorophyll fluorescence imaging, multicolor fluorescence imaging, plant phenotyping, drought stress, *Arabidopsis*, support vector machine

## Abstract

Plant responses to drought stress are complex due to various mechanisms of drought avoidance and tolerance to maintain growth. Traditional plant phenotyping methods are labor-intensive, time-consuming, and subjective. Plant phenotyping by integrating kinetic chlorophyll fluorescence with multicolor fluorescence imaging can acquire plant morphological, physiological, and pathological traits related to photosynthesis as well as its secondary metabolites, which will provide a new means to promote the progress of breeding for drought tolerant accessions and gain economic benefit for global agriculture production. Combination of kinetic chlorophyll fluorescence and multicolor fluorescence imaging proved to be efficient for the early detection of drought stress responses in the *Arabidopsis* ecotype Col-0 and one of its most affected mutants called *reduced hyperosmolality-induced [Ca^2+^]_i_ increase 1*. Kinetic chlorophyll fluorescence curves were useful for understanding the drought tolerance mechanism of *Arabidopsis*. Conventional fluorescence parameters provided qualitative information related to drought stress responses in different genotypes, and the corresponding images showed spatial heterogeneities of drought stress responses within the leaf and the canopy levels. Fluorescence parameters selected by sequential forward selection presented high correlations with physiological traits but not morphological traits. The optimal fluorescence traits combined with the support vector machine resulted in good classification accuracies of 93.3 and 99.1% for classifying the control plants from the drought-stressed ones with 3 and 7 days treatments, respectively. The results demonstrated that the combination of kinetic chlorophyll fluorescence and multicolor fluorescence imaging with the machine learning technique was capable of providing comprehensive information of drought stress effects on the photosynthesis and the secondary metabolisms. It is a promising phenotyping technique that allows early detection of plant drought stress.

## Introduction

Agricultural drought has become one of the major challenges in global agricultural production and food security. Plant responses to drought stress are complex, as plants varieties with different stress sensitivity might have different strategies for eliciting various mechanisms of drought avoidance and tolerance to maintain growth. Plant phenotypic traits related to morphological, physiological, and pathological traits are usually measured to evaluate the plant performance under different stresses. They are influenced by both environmental and genetic factors, and are essential in breeding research. There is an urgent need for developing advanced phenotyping techniques in drought-tolerant breeding programs.

*Arabidopsis* is a model system for dicot plant species with characteristics of small size, short life cycle, high viability, fertility, and small genome. Also, the whole genome sequencing of *Arabidopsis* was completed in 2000. *Arabidopsis* is used widely to study functional genomics and phenomics of plants. With phenotyping the model plant *Arabidopsis*, it is possible to benefit the progress of breeding and provide economic benefits for global crop production. However, traditional plant phenotyping methods are labor-intensive, time-consuming, and subjective, which becomes one of the major bottlenecks to exploit genetic information for genomic analysis (Rahaman et al., 2015).

Recently, various sophisticated imaging techniques have been integrated into advanced phenotyping platforms with the automatic control and data analysis system, which allows performing parallel studies of a large amount of plants and long-cycle growth monitoring under complex environments. Granier et al. (2006) evaluated drought tolerance of 9 *Arabidopsis* genotypes by estimating the rosette leaf area and the transpiration rate with a high-throughput phenotyping platform based on RGB imaging. Hyperspectral imaging was used to assess the rice growth and nitrogen status, which was useful for prediction of the rice yield and the grain quality (Nguyen and Lee, 2006). Zia et al. (2013) applied the thermography technique to evaluate three hundred genotypes of maize, and they demonstrated the negative correlation between the canopy temperature and the grain yield of the maizes at anthesis stage under drought-stressed condition. Due to a wide range of plant phenotypic traits, the fusion or integration of multiple sensors in one system was also used in various studies. For example, RGB imaging combined with kinetic chlorophyll fluorescence imaging could analyze plant growth, color, and photosynthetic traits of *Arabidopsis* under salt stress treatment simultaneously (Mariam et al., 2016). These non-destructive phenotyping techniques were also used to study the effects of biostimulates in terms of early detection of plant physiological stresses (Petrozza et al., 2014) or nutrient deficiency responses (Chaerle et al., 2007), dissection of the phenotypic trait components of plants with different genotypes underlying their growth and development (Chen et al., 2014), and selection of biotic and abiotic tolerant genotypes in crop improvement strategies (Mir et al., 2012).

Chlorophyll fluorescence imaging provides a powerful tool in plant phenotyping to image physiological phenomena interfered with photosynthetic apparatus and its associated metabolism (Humplik et al., 2015; Cen et al., 2017). There are three major types of fluorescence measurements to probe electron transfer reactions, including flash fluorescence measurement, saturating pulse and OJIP measurement and steady state measurement (Kalaji et al., 2014). Among them, kinetic chlorophyll fluorescence imaging adopts quenching kinetics and light curve protocol based on PAM, which can probe the performance of photosynthetic apparatus and evaluate the photosynthetic capacity. Serodio et al. (2017) assessed suspensions of the green microalga *Chlorella vulgaris* under the low and high light conditions via kinetic chlorophyll fluorescence imaging, providing a comprehensive description of photo-physiological response to light stress. Martinez-Ferri et al. (2016) used conventional chlorophyll fluorescence parameters to investigate the plant disease severity at the early stage, and they observed that *Fv’/Fm’* and *Fs/Fo* of avocado leaves decreased with the infection of white root rot. Plant defense responses to the biotic and abiotic stress factors not only reflect on the photosynthesis, but also on the secondary metabolites. Therefore, recent researches were also focused on exploring the multicolor fluorescence with the excitation of UV light. It usually obtains emission signals at red (690 nm) and far-red (740 nm) regions from chlorophyll *a*, and blue (440 nm) and green (520 nm) regions from plant phenolic compounds. Previous studies have successfully applied multicolor fluorescence for evaluating fruit qualities (Lichtenthaler et al., 2012), pathogen attack (Ortiz-Bustos et al., 2016; Perez-Bueno et al., 2016), and nutrient and water deficiencies (Hsiao et al., 2010; Tremblay et al., 2012). However, there were few studies combining kinetic chlorophyll fluorescence and multicolor fluorescence imaging techniques as a means to study phenotyping of plant response to drought stress. Furthermore, these methods were generally employed in fundamental research by using limited fluorescence parameters, and the fundamental information hidden in the fluorescence images has not been thoroughly investigated (Woo et al., 2008; Bresson et al., 2015).

Hence, this study was aimed to investigate the feasibility of applying kinetic chlorophyll fluorescence and multicolor fluorescence imaging for plant phenomics in terms of drought stress response. Advanced machine learning methods were also proposed to classify the healthy and the drought-stressed plants at different stages. In turn, this could be integrated into a large-scale plant phenotyping pipeline, and eventually speed up the development process of breeding drought-resistant crops.

## Materials and Methods

### Plant Growth Condition and Drought Treatment

**FIGURE 1:**
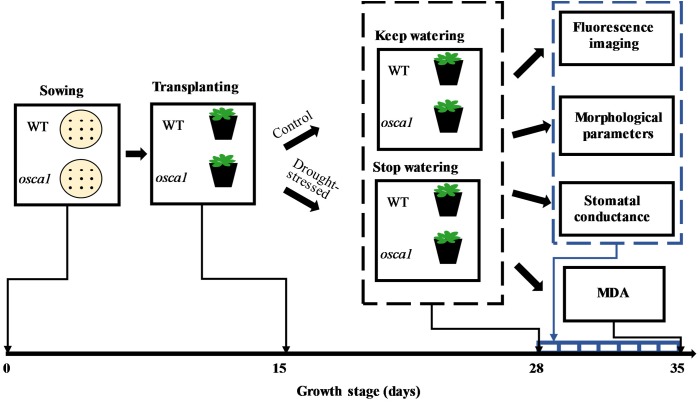
Protocol of plant drought stress treatment during growth stage. WT, *osca1*, and MDA represent wild-type, *reduced hyperosmolality-induced [Ca2+]i increase 1*, and malondialdehyde, respectively.

The *Arabidopsis* ecotype Col-0 (pMAQ2) and one of its most affected mutants called *reduced hyperosmolality-induced [Ca^2+^]_i_ increase 1* (*osca1*) as reported by Yuan et al. (2014) were provided by Plant Environmental Sensing Laboratory of Hangzhou Normal University. The plant treatment protocol is presented in **Figure 1**. Cultivation method of *Arabidopsis* followed Yuan et al. (2014) with minor modification. Briefly, seeds of each genotype were sown on Petri dishes in half-strength Murashige and Skoog salts, 1% (w/v) sucrose, and 0.8% (w/v) agar. At four-leaf stage (day 15 after sowing), healthy plants with a similar growth status were transplanted into 210 mL pots with three plants in each. Pots were filled with a mixture of soil, perlite, and vermiculite (v: v: v = 2:1:1), and 15 mL water with 0.1% nutrient solution initially. Plants were cultivated in a growth chamber (AR-41l2, Percival Scientific, Perry, GA, United States) with the temperature at 22°C, the relative humidity at 65%, and the photoperiods of 16/8 h light/dark by using the 100 μmol m^-2^ s^-1^ cool-white fluorescent illumination. Plants were watered with 6 ml 1% nutrient solution every day. At ten-leaf stage (day 28 after sowing), 59 pots of each genotype were withheld from watering for 8 days as the drought stress treatment based on the preliminary experiment, and the other 59 pots of each genotype continued to be watered daily during the whole experiment as the control. A total of 236 pots were used in this experiment with 208 pots for non-destructive fluorescence imaging, and 28 pots for destructive measurement of the leaf stomatal conductance. Meanwhile, 96 pots randomly selected from 208 pots were used for the measurements of morphological parameters and malondialdehyde (MDA).

### Kinetic Chlorophyll Fluorescence and Multicolor Fluorescence Imaging

A PAM fluorescence imaging system (FluorCam FC 800, Photon Systems Instruments, Brno, Czechia) was used to obtain the kinetic chlorophyll fluorescence curves and images of plants every day upon the onset of drought stress. The system consisted of a high-speed CCD camera with the spatial resolution of 1392 pixels × 1040 pixels, a prime lens (SV-H1.4/6, VS Technology, Tokyo, Japan), four LED panels, and a sample handling unit with a manually adjustable vertical stage. The light source included two orange-red LED panels for MF (MF, a very weak intensity of short flash, <0.1 μmol m^-2^ s^-1^) and AL 1 (strong intensity continuous light, 0–250 μmol m^-2^ s^-1^), and two cool-white LED panels for SF (0–3000 μmol m^-2^ s^-1^) and AL 2 (strong intensity continuous light, 0–1600 μmol m^-2^ s^-1^), with the angle of 45°. The distance between the plant canopy and the lens was 22 cm. After dark adaptation for 20 min until the PSII reaction centers opened, the kinetic chlorophyll fluorescence curves and images of plants were acquired with an optimized quenching kinetic protocol as follows. Plants were first exposed to the dark for 17 s. In the dark environment, *Fo* was measured with a 4040 ms flash of light. *Fm* was then measured at 5.56 s with a 320 ms saturating flash at ∼2300 μmol m^-2^ s^-1^, followed by exposing plants to actinic light at a constant of 100 μmol m^-2^ s^-1^ for 70 s. *Fp* was measured at 23.12 s. *Ft_Ln* was measured before any of the saturating flash, and later, the signal declined to *Ft_Lss*. *Fm_Ln* and *Fm_Lss* were measured with the SF at 32.24, 42.24, 52.24, 72.24, and 92.24 s. By following the actinic light period, fluorescence signals during dark relaxation were recorded by measuring *Ft_Dn*, and acquiring *Fm_Dn* with the SF at 122.24, 152.24, and 182.24 s. Based on the basic chlorophyll fluorescence signals, the maximum quantum yield of PSII photochemistry (*Fv/Fm*), instantaneous non-photochemical quenching during light adaptation (*NPQ_Ln*), steady-state non-photochemical quenching (*NPQ_Lss*), instantaneous fluorescence decline ratio in light (*Rfd_Ln*), steady-state PSII quantum yield (Φ*PSII_Lss*), PSII quantum yield during light adaptation (Φ*PSII_Ln*), coefficient of non-photochemical quenching during dark relaxation (*qN_Dn*), coefficient of non-photochemical quenching during light adaptation (*qN_Ln*), coefficient of photochemical quenching during light adaptation (*qL_Ln*), coefficient of photochemical quenching in steady-state (*qL_Lss*), variable fluorescence in steady-state (*Fv_Lss*), and variable fluorescence during light adaptation (*Fv_Ln*) can be derived as equations (1–10) (Murchie and Lawson, 2013):

(1) Fv/Fm = (Fm − Fo)/Fm

(2) NPQ_Ln = (Fm − Fm_Ln)/Fm_Ln

(3) NPQ_Lss = (Fm − Fm_Lss)/Fm_Lss

(4) Rfd_Ln = (Fp − Ft_Ln)/Ft_Ln

(5) ΦPSII_Lss = (Fm_Lss − Ft_Lss)/Fm_Lss

(6) ΦPSII_Ln = (Fm_Ln − Ft_Ln)/Fm_Ln

(7) qN_Dn = (Fm− Fm_Dn)/(Fm − Fo_Dn)

(8) qN_Ln = (Fm− Fm_Ln)/(Fm − Fo_Ln)

(9) $$\begin{array}{lll} qL\_Ln\;\; & = & \left(Fm\_Ln-\;Ft\_Ln)/(Fm\_Ln\;-\;Fo\_Ln\right)\\ & & /(Fo\_Ln/Ft\_Ln) \end{array}$$

(10) $$\begin{array}{lll} qL\_Lss\;\; & = & \left(Fm\_Lss-\;Ft\_Lss)/(Fm\_Lss\;-\;Fo\_Lss\right)\\ & & /(Fo\_Lss/Ft\_Lss) \end{array}$$

where *n* is related to the moments of irradiance of suturing flashes. Finally, a total of 89 chlorophyll fluorescence images were obtained based on this protocol.

For multicolor fluorescence image acquisition, a UV (320–400 nm) LED panel was used as the excitation source. Multicolor fluorescence images in blue (440 nm), green (520 nm), red (680 nm), and far-red (740 nm) wavelength regions excited by UV light were acquired sequentially. In order to improve the signal-to-noise ratio, five images with the exposure time of 4 s were averaged to calculate *BF*, *GF*, *RF*, and *IrF* for each measurement, and a total of 16 multicolor fluorescence images were obtained.

### Measurement of Plant Morphological and Physiological Parameters

In order to evaluate the effect of drought stress on the plant morphological and physiological characteristics, and to determine the most useful phenotypes to understand the early response to drought stress, parameters including projected leaf area, rosette leaf number, plant height, flowering plant number, leaf stomatal conductance, and MDA were determined after performing fluorescence imaging.

The projected leaf area was calculated from the total pixels of the chlorophyll fluorescence image. The total rosette leaf number was counted every day after drought stress. The plant height was measured from the base of the stem to the tip of the main inflorescence of the plant from day 3 to day 8 after drought stress, and at the same time, the flowering plant number was also recorded by counting the new flowering plant.

Leaf stomatal conductance was measured using a leaf porometer (SC-1, Decagon Devices, Pullman, WA, United States) after performing the calibration with a filter paper of 100% humidity as a reference (according to the user menu). The rosette leaves of the plants cannot be measured directly due to the size of leaves. Therefore, nine rosette leaves were randomly cut from one pot of each genotype, and then leaves of each treatment were examined immediately. Since leaves were still small at day 1 and also drought-stressed leaves wilted at day 7, the stomatal conductance was only recorded from day 2 to 6 after drought stress.

Malondialdehyde was determined by the thiobarbituric acid (TBA) method (Sunkar et al., 2003). Leaves of six biological replicates (four pots for each) from each wild-type or *osca1* plants were selected for MDA measurement at the last day of the experiment (day 8 after drought stress). 0.1 g leaf tissue of each repetition was homogenized in 5 mL of 0.1% trichloroacetic acid (TCA), and the homogenate was centrifuged (Neofuge 15R, Heal Force, Shanghai, China) at 10,000 × *g* for 5 min at 4°C. 20% TCA containing 0.5% TBA was added to 1 mL supernatant. The mixture was then boiled for 30 min at 95°C, followed by the centrifugation at 10,000 × *g* for 10 min. The absorbance of the supernatant was measured at 532 nm and 600 nm with a microplate spectrophotometer (Epoch 2, BioTek Instruments, Winooski, VT, United States). MDA content was then calculated using an extinction coefficient of 155 Mm^-1^ cm^-1^ (Heath and Packer, 1968).

### Data Analysis

A total of 105 fluorescence images including 89 chlorophyll fluorescence images and 16 multicolor fluorescence images were acquired from each pot. The image preprocessing was performed including the selection of ROIs, background segmentation, and computation of mean fluorescence parameters from the corresponding ROIs. After preprocessing, ANOVA was implemented first to identify the differences between the control and the drought stress treatments among 5 morphological and physiological related parameters, 4 commonly used chlorophyll fluorescence parameters, and 4 basic multicolor fluorescence parameters. To further explore fluorescence parameters, feature selection by using SFS algorithms with Fisher criterion was then performed to extract the most important features that could differentiate the drought-stressed plants from the control. SFS algorithm is a bottom-up procedure that starts with an empty subset of features and repeatedly adds one new feature with the best function criterion value in the subset each time (Cen et al., 2017). This procedure will not be stopped until a predefined number of features are selected with highest classification accuracy. In this study, 9 features were obtained from 105 fluorescence parameters in both wild-type and *osca1*, and Pearson’s correlations among fluorescence, morphological and physiological traits were visualized in a network to gain a better understanding of the phenotypic relationship.

In order to differentiate the drought-stressed plants from the control based on the selected features, SVM was used for the two-class classification. The SVM classifier can separate a given set of labeled training data with a hyper-plane that is maximally distant from the training data set. It employs a linear kernel function to solve a convex quadratic programming problem (Furey et al., 2000). The two-class classifications with labeling “1” for the control plants and “2” for the drought-stressed ones were performed at day 1, 3, 5, 7, and 8, respectively. For both classification schemes of two genotypes, the data set of 108 pots were divided into two groups with 9/10 samples for the training set and 1/10 samples for the testing set with ten repetitions by using 10-fold cross-validation (Aidan and Nicholas, 2006). In this study, ANOVA and Pearson’s correlation coefficient were carried out using IBM SPSS Statistics 19 (IBM Corporation, United States). Image data analyses including preprocessing, feature selection, and classification were performed in Matlab R2014a (MathWorks, United States) and Python 3.4 (Python Software Foundation, United States) with open source scikit-learn^1^.

## Results

### Effect of Drought Stress on Morphological and Physiological Traits Over Time

**FIGURE 2:**
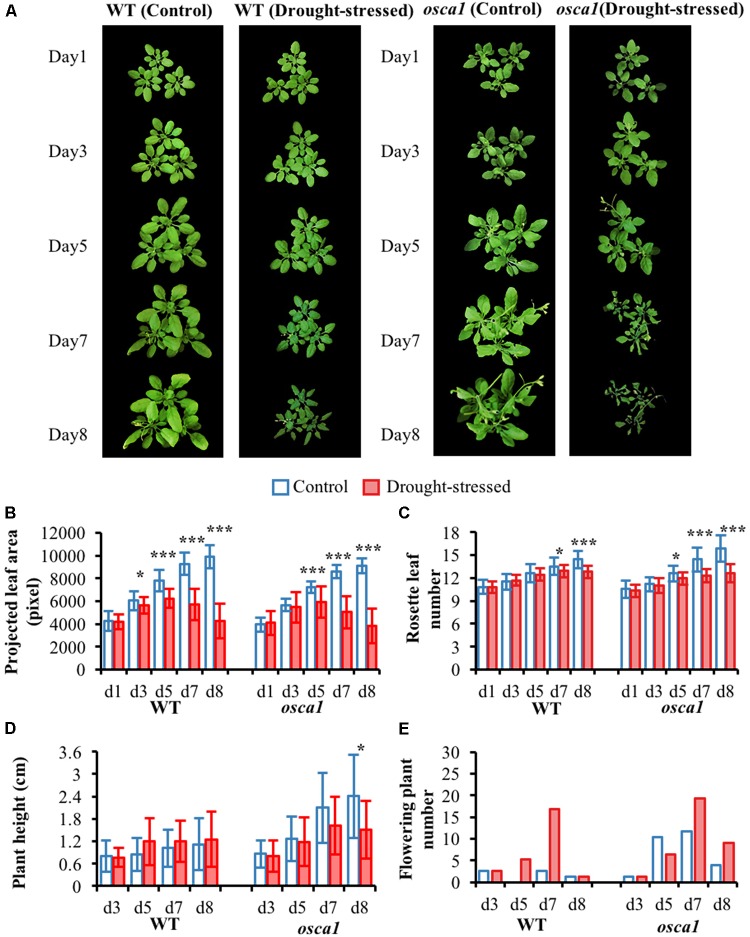
**(A)** RGB images, and morphological parameters including **(B)** projected leaf area, **(C)** rosette leaf number, **(D)** plant height, and **(E)** flowering plant number of wild-type (WT) and *reduced hyperosmolality-induced [Ca^2+^]_i_ increase 1* (*osca1*) under two treatments over time. Values shown in **(B–D)** are mean values and standard deviations, with statistically significant differences indicated (^∗^*P* < 0.05; ^∗∗^*P* < 0.01; ^∗∗∗^*P* < 0.001). Values shown in the **(E)** are total new flowering plants that counted from 52 pots.

The RGB images and the morphological parameters of wild-type and *osca1* under control and drought stress treatments at day 1, 3, 5, 7, and 8 after exposure to the drought stress are presented in **Figure 2**. The parameters included projected leaf area, rosette leaf number, plant height, and flowering plant number. It was visually observed that both wild-type and *osca1* presented wilting leaves under the drought-stressed condition. In general, the mutant *osca1* wilted more seriously than wild-type. The investigation of morphological traits including the projected leaf area and the rosette leaf number indicated that low water availability had significant effects on the plant growth (**Figures 2B,C**). The projected leaf area of wild-type and *osca1* decreased evidently after 3–8 days and 5–8 days post-drought treatment, respectively. While the significant decrease in the rosette leaf number with drought stress was observed at day 7 in wild-type and at day 5 in *osca1*. Drought stress had no significant effect on the plant height except for *osca1* at day 8 (**Figure 2D**). The average plant height of *osca1* was higher than wild-type in the control condition. The increased flowering plants of wild-type and *osca1* under drought treatment compared to the control treatment indicated earlier flowering time with the drought stress (**Figure 2E**).

**FIGURE 3:**
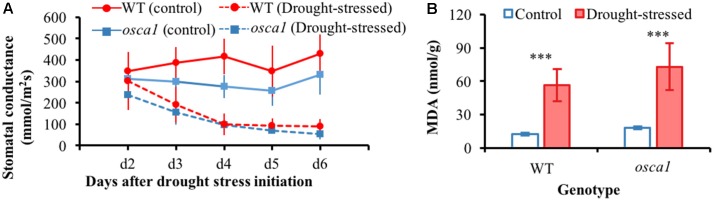
**(A)** Stomatal conductance from day 2 to 6 for wild-type (WT) and *reduced hyperosmolality-induced [Ca^2+^]_i_ increase 1* (*osca1*) plants grown in two treatment conditions, and **(B)** malondialdehyde (MDA) contents of the control and drought-stressed plants at day 8. Error bars represent the standard deviations, and statistically significant differences are indicated (^∗∗∗^*P* < 0.001).

**Figure 3A** presents stomatal conductance of wild-type and *osca1* plants under the drought stress condition as well as the control condition. There is a clear decrease in the stomatal conductance values in both wild-type and *osca1* plants with the drought stress treatment over time. MDA contents of the control and the drought-stressed plants at day 8 are shown in **Figure 3B**, which is considered as a reliable indicator of lipid membrane peroxidation (Sperdouli and Moustakas, 2012). MDA levels for both genotypes increased dramatically after 8 days exposure to drought stress, and higher values in MDA were obtained in *osca1*, suggesting a more severe degree of lipid peroxidation.

### Kinetic Chlorophyll Fluorescence Curve Captured Early Responses to Drought Stress

**FIGURE 4:**
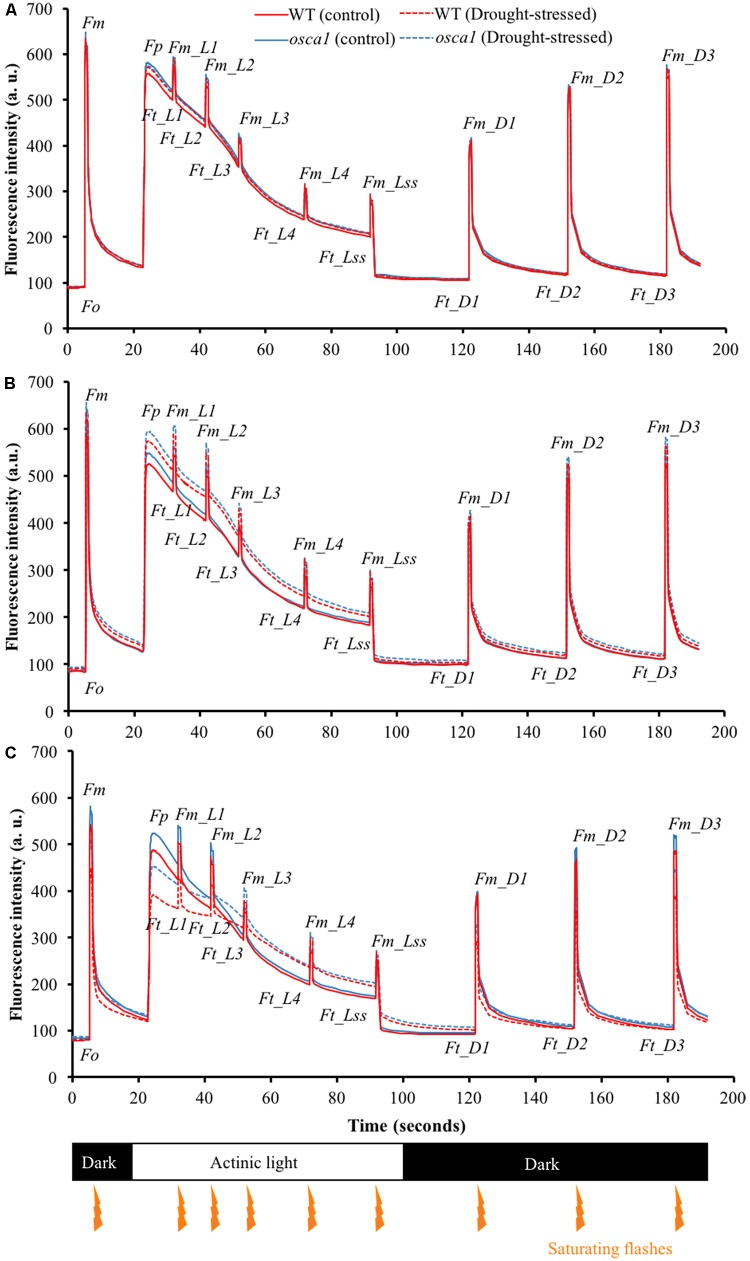
Representative kinetic chlorophyll fluorescence curves of wild-type (WT) (blue lines) and *reduced hyperosmolality-induced [Ca^2+^]_i_ increase 1* (*osca1*) (red lines) plants in the control (solid lines) and the drought-stressed (dashed lines) treatments at day 1 **(A)**, day 3 **(B)**, and day 7 **(C)** after introducing the drought stress. Orange arrows indicate the timings of the saturating flashes, and values represent the average of three replicates per genotype and treatment. Please refer to “Abbreviations” for all the fluorescence parameters.

To understand the photosynthetic performance of the control and the drought-stressed plants, chlorophyll fluorescence curves were measured from day 1 to day 8 under drought stress by using the chlorophyll fluorescence quenching protocol (Cen et al., 2017). Typical kinetic chlorophyll fluorescence curves of control and drought-stressed plants at day 3 are presented in **Figure 4**. Day 3 was the earliest day that could visually observe the pattern differences of the kinetic chlorophyll fluorescence curves between the drought stress treatment and the control for both genotypes. It was observed that the parameters of *Fp*, *Ft_Ln*, *Fm_Ln* measured from the drought-stressed plants increased rapidly compared with those of the control, while the changes in *Ft_Dn* and *Fm_Dn* were less pronounced. In addition, the upward shifts of the drought-stressed plants could be detected as early as day 1 for *osca1* but day 3 for wild-type (**Figures 4A,B**). The slopes of the curves from *Fp* to *Fm_L2* for both genotypes reduced significantly after 7 days exposure to drought stress (**Figure 4C**), indicating the slow fluorescence quenching process.

### Kinetic Chlorophyll Fluorescence Imaging Indicated the Process of Response to Drought Stress

**FIGURE 5:**
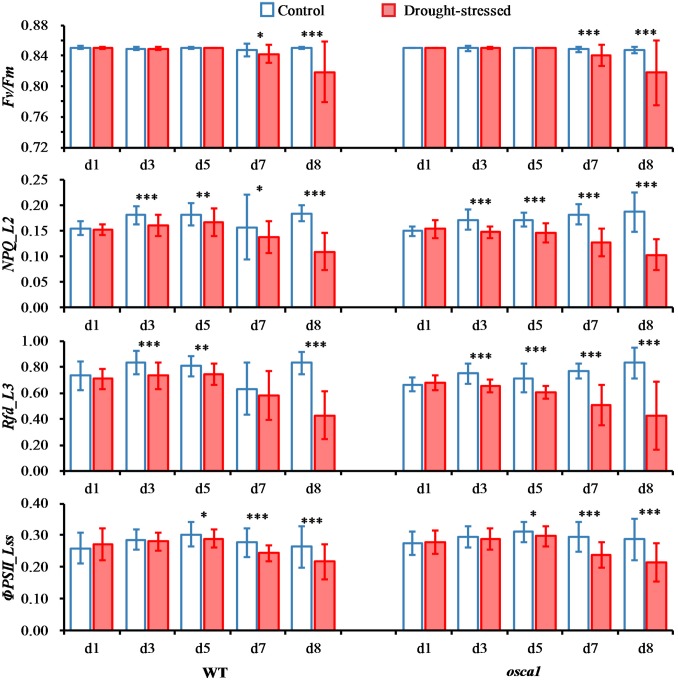
Means and standard deviations of commonly used chlorophyll fluorescence parameters of wild-type (WT) and *reduced hyperosmolality-induced [Ca^2+^]_i_ increase 1* (*osca1*) plants under two treatments over time. The chlorophyll fluorescence parameters include maximum quantum yield of PSII photochemistry for the dark-adapted (*Fv/Fm*), non-photochemical quenching during light adaptation (*NPQ_L2*), fluorescence decrease ratio during light adaptation (*Rfd_L3*), and steady-state PSII quantum yield (Φ*PSII_Lss*). Statistically significant differences are indicated (^∗^*P* < 0.05; ^∗∗^*P* < 0.01; ^∗∗∗^*P* < 0.001).

Commonly used chlorophyll fluorescence parameters averaged from ROIs were also investigated to further explore the plant response to drought stress on photosynthetic performance, including *Fv/Fm, NPQ_L2, Rfd_L3*, and Φ*PSII_Lss* (**Figure 5**). Overall, wild-type and *osca1* had similar patterns under the control and the drought conditions. The mean values of *Fv*/*Fm* in control plants were relatively consistent with the values at 0.85 ± 0.002 over time, while significant decreases in drought-stressed plants were observed at day 7 (*P* < 0.05) and day 8 (*P* < 0.001) with the values at 0.84 ± 0.01 and 0.82 ± 0.04 for wild-type and *osca1*, respectively. *NPQ_L2* of both genotypes showed significant decreases in the drought stress treatment starting from day 3 compared with the control set, indicating that this parameter was sensitive to drought stress. Similar patterns were also found in *Rfd_L3* except wild-type at day 7. The values of Φ*PSII_Lss* in all the treatment groups increased from day 1 to 5 and then decreased. The significant difference between the control and the drought stress treatment was detected at day 5 for both genotypes.

**FIGURE 6:**
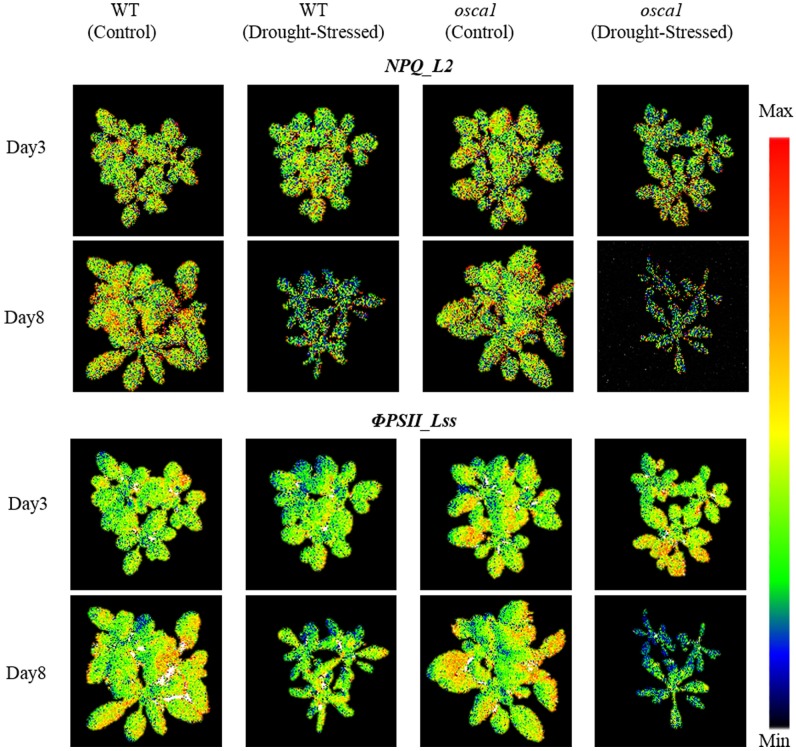
Representative chlorophyll fluorescence images of non-photochemical quenching during light adaptation (*NPQ_L2*) and steady-state PSII quantum yield (Φ*PSII_Lss*) for wild-type (WT) and *reduced hyperosmolality-induced [Ca^2+^]_i_ increase 1* (*osca1*) plants under the control and the drought treatment at day 3 and 8 after drought stress initiation. The color code depicted at the right of the images ranges from black (minimum value) to red (maximum value).

The parameters of chlorophyll fluorescence only showed the average trends of the canopy without reflecting the spatial variation. **Figure 6** presents representative chlorophyll fluorescence images, including *NPQ_L2* and Φ*PSII_Lss*, according to the parameters that have the potential to explore the spatial heterogeneity. The intensity of *NPQ_L2* in the drought stress treatment decreased compared with the control as early as day 3, while the spatial variation was not obvious. Images of Φ*PSII_Lss* decreased compared with the control as early as day 3, and this trend spread from the leaf tips and edges to the entire canopies indicating the spatial heterogeneity.

### Multicolor Fluorescence Imaging Indicated the Effect of Drought Stress on Plant Metabolism

**FIGURE 7:**
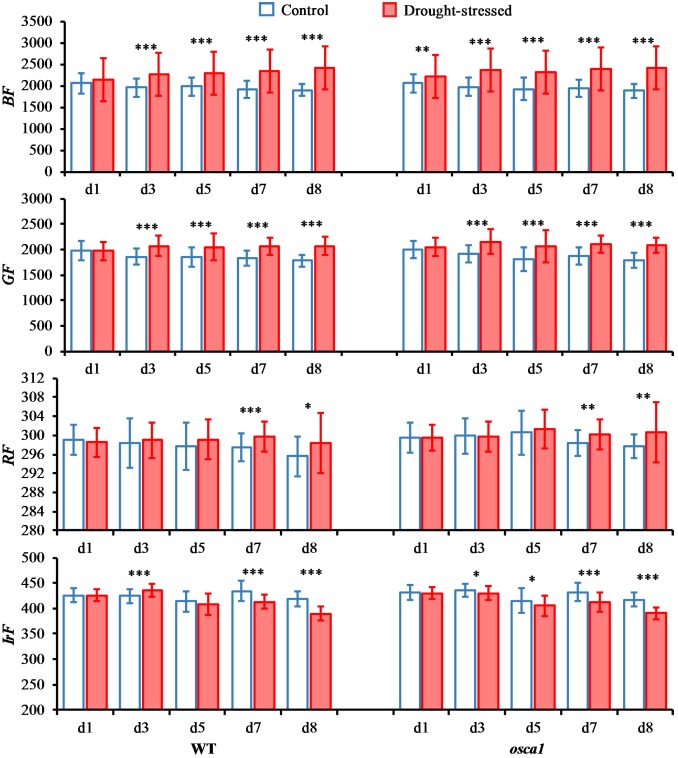
Means and standard deviations of basic multicolor fluorescence parameters, including blue fluorescence (*BF*), green fluorescence (*GF*), red fluorescence (*RF*), and far-red fluorescence (*IrF*), of wild-type (WT) and *reduced hyperosmolality-induced [Ca^2+^]_i_ increase 1* (*osca1*) plants under two treatments over time. Statistically significant differences are indicated (^∗^*P* < 0.05; ^∗∗^*P* < 0.01; ^∗∗∗^*P* < 0.001).

**FIGURE 8:**
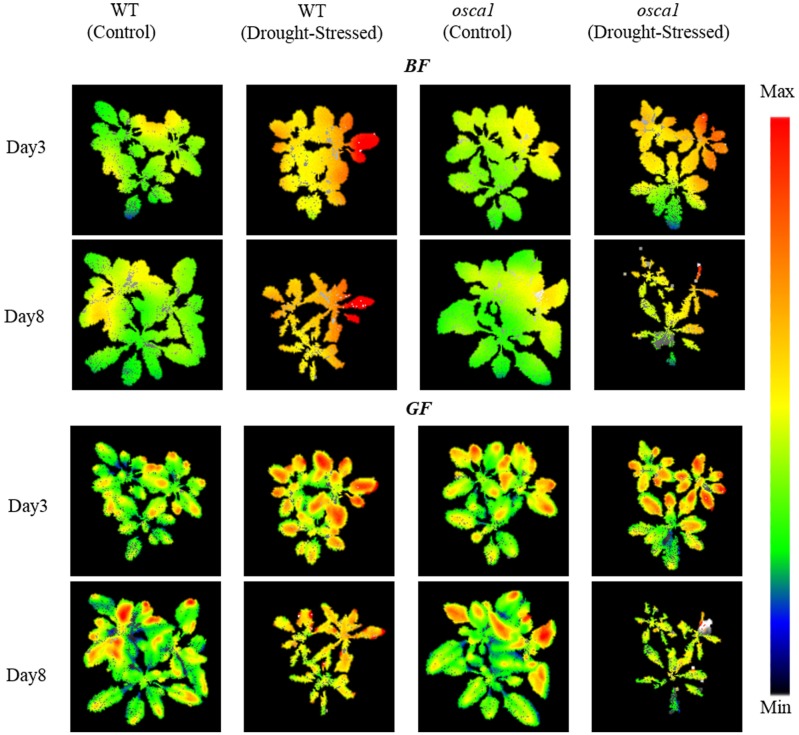
Representative multicolor fluorescence images of *BF* (440 nm) and *GF* (520 nm) for wild-type (WT) and *reduced hyperosmolality-induced [Ca^2+^]_i_ increase 1* (*osca1*) plants in control and drought stress treatments at day 3 and 8 after drought stress initiation. The color code depicted at the right of the images ranges from black (minimum value) to red (maximum value).

Basic multicolor fluorescence parameters including *BF*, *GF*, *RF*, and *IrF* were selected from the full data set (**Figures 7**, **8**). *RF* and *IrF* are emitted by chlorophyll *a*, and *BF* and *GF* are emitted by phenolics, which could provide information related to the primary and secondary metabolism, respectively (Pineda et al., 2008). The *BF* values for the control set of both genotypes were relatively consistent during plant growth, while the values showed significant increases starting from day 3 in wild-type and day 1 in *osca1* after drought stress treatment. The *GF* values of both genotypes in the control groups decreased slightly during plant growth, while the *GF* of both genotypes increased significantly in the drought stress treatment from day 3 to 8 compared with that of the control treatment. In contrast, the parameter *RF* was not as sensitive as *BF* and *GF* to the drought stress. No significant differences were observed until day 7 and 8 in wild-type and *osca1*. The drought stress generally caused the decreases in *IrF* for both wild-type and *osca1*, and the declined trends of drought-stressed plants over time were also observed.

The representative *BF* and *GF* images at day 3 and 8 after drought stress treatment as well as the control sets are shown in **Figure 8**. The increase in *BF* after drought stress appeared in the entire canopy for both genotypes, while the increased signals of *GF* for the drought-stressed plants spread from the middle to the edge of the leaves causing the spatial heterogeneities within the leaf and the canopy level.

### Classification Models for Drought Stress Detection

**FIGURE 9:**
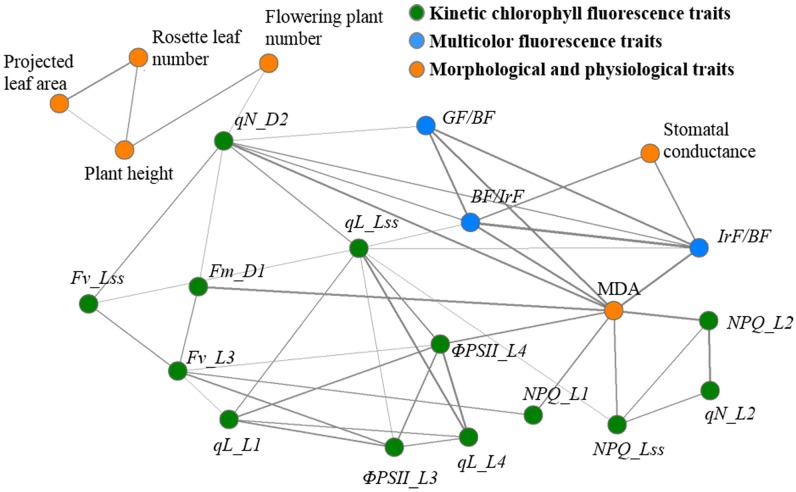
Network visualization of significant phenotypic correlations between the 16 fluorescence traits (kinetic chlorophyll fluorescence traits, green nodes; multicolor fluorescence traits, blue nodes) and 6 morphological and physiological traits (orange nodes). For visualization purpose, only significant correlations are shown (*P* < 0.01 and |*r*| > 0.40). Correlations are shown by solid lines, which are associated with tie strength. Please refer to “Abbreviations” for all the fluorescence parameters.

Although commonly used fluorescence parameters and images can provide a basic evaluation of plant phenotypes of the drought stress response, it is difficult to perform a quantitative analysis to clearly differentiate the drought-stressed plants from the normal ones. Therefore, machine learning techniques were employed to further explore the fluorescence images. The optimum feature subsets of two genotypes selected by SFS were fluorescence far-red/blue ratio (*IrF/BF*), *qN_D2*, *Fv_Lss*, *qL_L4*, *qN_L2*, Φ*PSII_L4*, *qL_L1*, *NPQ_L1*, and *Fm_D1* for wild-type, and fluorescence blue/far-red ratio (*BF/IrF*), *NPQ_L2*, *NPQ_Lss*, fluorescence green/blue ratio (*GF/BF*), *Fv_L3*, *qN_D2*, *qL_Lss*, Φ*PSII_L3*, and Φ*PSII_L4* for *osca1*. Trait–trait correlations among the total 16 selected fluorescence, 4 morphological and 2 physiological traits were shown in a correlation network (**Figure 9**), which presented phenotypic similarity of traits related to the drought stress response. Traits of the same categories showed good correlations (|*r*| > 0.40, *P* < 0.01) with each other. Physiological traits correlated well with fluorescence traits. MDA and stomatal conductance displayed high correlations with fluorescence traits, and MDA had the highest correlation with the fluorescence trait of *NPQ_L2* (|*r*| = 0.87, *P* < 0.01) among the selected 16 fluorescence traits while stomatal conductance had the highest correlation with *IrF/BF* (|*r*| = 0.67, *P* < 0.01). However, the correlation between morphological traits and fluorescence traits were not significant. Flowering plant number only correlated with *qN_D2* (|*r*| = 0.41, *P* < 0.01). Projected leaf area, rosette leaf number, and plant height showed poor correlation with fluorescence traits (|*r*| < 0.40, *P* < 0.01).

Most of the fluorescence parameters showed a significant difference between the drought-stressed plants and the control ones. The classification models at different drought stress levels were then constructed with the SVM classifier using the fluorescence features selected by SFS, and the results were summarized in **Table 1**. The classification accuracies of wild-type and *osca1* reached 87.5 and 93.3%, respectively, at day 3 after drought stress initiation. The drought stress treatment induced the changes of kinetic chlorophyll fluorescence and multicolor fluorescence, and their sensitivities to the drought stress varied with the drought stress time and the growth stage. At the late stage, 8 days after drought stress, the classification accuracies in both genotypes reached 99.1%.

**Table 1:** Support vector machine (SVM) classification results of discriminating control and drought-stressed wild-type (WT) and *reduced hyperosmolality-induced [Ca^2+^]_i_ increase 1* (*osca1*) plants based on the nine traits of kinetic chlorophyll fluorescence and multicolor fluorescence selected by sequential forward selection (SFS).

Drought-stressed time	Genotype					
	WT			*osca1*		
	Control (%)	Drought-stressed (%)	Overall accuracy (%)	Control (%)	Drought-stressed (%)	Overall accuracy (%)
Day 1	55.7	71.1	63.4	75.0	73.1	74.1
Day 3	94.2	80.8	87.5	96.2	90.4	93.3
Day 5	100.0	84.6	92.3	98.1	84.6	91.4
Day 7	100.0	98.1	99.1	100.0	98.1	99.1
Day 8	100.0	98.1	99.1	100.0	98.1	99.1

## Discussion

Plants manifest multiple defense mechanisms involving various morphological and physiological alterations in response to drought stress (Joshi et al., 2016). Investigation of RGB images (**Figure 2A**) revealed that leaf rolling followed by wilting might be an initial morphological survival mechanism against drought stress that could reduce the transpiration rate, thereby improving water use efficiency. The decline in the projected leaf area and the leaf number demonstrated the progress of plant senescence or damages under drought stress. Both genotypes of *Arabidopsis* conferred a drought escape strategy of early flowering during the reproductive stage (**Figure 2E**), which could ensure their survival under drought stress. Earlier flowering of *osca1* in drought stress treatment implied its sensitivity to drought stress and shorter survival compared to wild-type under drought stress (Kazan and Lyons, 2016). These changes in morphological traits not only indicated that *osca1* displayed defects in major aspects of the osmotic stress signaling pathway, but also implied that OSCA1, a channel responsible for [Ca^2+^]_i_ increases induced by a stimulus in plants, might play a key role in responding to drought stress at the whole-plant level (Yuan et al., 2014). Stomatal closure is one of the most important responses to drought stress by reducing water loss and increasing the water use efficiency (Farooq et al., 2009). The stomata stayed more open in *osca1* than wild-type under drought stress treatment (**Figure 3A**), which was consistent with the study reported by Yuan et al. (2014), suggesting that the mutant *osca1* with impaired OSCA1 might not induce the accumulation of abscisic acid (ABA) trigger stomatal closure at time. Previous studies have demonstrated that the activity of antioxidant enzymes was correlated with plant tolerance to abiotic stresses, such as drought stress, and MDA was the final product of plant cell membrane lipid peroxidation and one of the important signs of membrane system injury (Sperdouli and Moustakas, 2012; Mirzaee et al., 2013). In this study, *osca1* had a higher MDA level (**Figure 3B**) after 8 days post-drought stress indicating a higher sensitivity to drought stress, which agreed with the findings of Siddiqui et al. (2015).

Drought stress is one of the most severe limitations to photosynthesis, which causes the diffusion limitations through the stomata and the photosynthetic metabolism. Kinetic chlorophyll fluorescence imaging is a useful tool to monitor subtle changes in the photochemical and non-photochemical process. Different patterns of chlorophyll fluorescence quenching curves implied that wild-type and *osca1* exhibited different photosynthetic strategies to utilize the absorbed irradiance under drought stress. Wild-type can efficiently keep homeostasis of photosynthetic apparatus and the plants under the drought-stressed treatment yield almost similar chlorophyll fluorescence transients to the control at the early stage of drought stress (**Figure 4A**). At the later stage when plant leaves turned wilted, the results of decreased patterns of drought stress treatment were similar to the findings of Mishra et al. (2014). This indicated a perturbation in PSII functional properties.

Conventional fluorescence parameters including *NPQ_L2*, *Rfd_L3*, and Φ*PSII_Lss* were considered as straightforward and practical parameters to monitor the response to drought stress (**Figure 5**). *Fv/Fm* of both genotypes were not significantly different between the control and drought stress treatments until 7 days’ drought stress, indicating that *Fv/Fm* might not be a good indicator of short-term plant drought stress. *Rfd_L3* presented the potential photosynthetic capacity of plants. The *Rfd_L3* of drought-stressed groups of two genotypes showed a declining pattern after drought stress induction, suggesting that *Rfd_L3* was more sensitive to drought stress than *Fv/Fm* (Lichtenthaler et al., 2005). The *NPQ_L2* can be used to assess a photo-protective process, reflecting the adaptation of plants to stress environments (Murchie and Lawson, 2013). The *NPQ_L2* values of drought-stressed wild-type and *osca1* plants decreased at day 7 and day 8, indicating that senescence processes caused incapacity of protection mechanism process for down-regulation (Gorbe and Calatayud, 2012). The parameter Φ*PSII_Lss* measures the efficiency of PSII photochemistry, and presents the proportion of the light absorbed by chlorophyll associated with PSII that is used in photochemistry. A significant decrease in Φ*PSII_Lss* of wild-type and *osca1* indicated that the severe stomatal closure reduced the CO_2_ supply to chloroplasts (Zhou et al., 2017). Additionally, the chlorophyll fluorescence images could display the spatial variations in the whole canopy and explain the spatial heterogeneity during drought stress. Different fluorescence signals of leaf tips and edges at the whole canopy might imply a greater potential restriction in photosynthesis and suppression of the protective ability in these areas.

The multicolor fluorescence imaging is also considered as a useful tool to detect drought stress response in physiology (Pineda et al., 2008). Drought stress treatment in both genotypes caused an increase in *BF* and *GF* (**Figure 7**), which was in agreement with a reported study of tobacco (Lang et al., 1996). This might have been caused by the accumulation of blue-green fluorescence emitting substances induced by drought stress, which were known to be synthesized as intermediates during chlorophyll breakdown. Previous studies also reported that the BF and GF could be primarily emitted from the epidermis, the mesophyll cell walls and the leaf veins by several cell wall-bound phenolic compounds (Pineda et al., 2008; Perez-Bueno et al., 2016). But there is a lack of BF and GF signal analysis in drought-stressed plants. Meanwhile, the wilting of leaves under drought stress also caused a range of optical changes resulting in an increased reflectance in the visible range. The increase of *RF* in both genotypes under drought stress compared to the control set (**Figure 7**) might be a result of the gradual loss of photosynthetic quantum conversion which could increase the red chlorophyll fluorescence yield or the breakdown of chlorophylls that the plants suffered a period of lack of water (Gitelson et al., 1998). However, *IrF* of wild-type and *osca1* under drought stress decreased in comparison with those of the control set (**Figure 7**), due to the lack of re-absorption of the emitted chlorophyll fluorescence band *in vivo* chlorophyll protein complexes (Lichtenthaler et al., 2012).

The SVM classifier combined with the SFS feature selection method has shown a good classification performance. Although fluorescence parameters were not well correlated with the morphological parameters based on Pearson’s correlation analysis, the correlation network showed trait similarity of the same categories and high correlations between fluorescence parameters and physiological parameters (**Figure 9**), indicating that SFS feature selection methods could provide valid information related to the drought stress response with only a few number of traits. Additionally, it was noted that the classification accuracies of both genotypes was 87.5% at least for the plants as early as day 3 after drought stress treatment, which revealed that the drought stress could be detected by the fluorescence imaging at the early stage before the wilted symptoms appeared. Advanced machine learning methods showed superior ability to detect drought stress response. The classification accuracy of *osca1* was higher than that of wild-type at day 3, which indicated a higher sensitivity of *osca1* to drought stress.

This study has shown the potential of kinetic chlorophyll fluorescence and multicolor fluorescence imaging for the early detection of drought stress responses in two different genotypes of *Arabidopsis*. The kinetic chlorophyll fluorescence curves have the potential to detect the drought tolerance mechanism of *Arabidopsis*. Even though some conventional fluorescence parameters provided qualitative information related to the drought stress response in different genotypes including the spatial heterogeneities of drought stress response within the leaf and the canopy level, the selected fluorescence parameters by SFS presented a higher correlation with physiological traits, and they have shown a better result to classify the drought-stressed and the control plants by using SVM classifier. The results demonstrated that the combination of kinetic chlorophyll fluorescence and multicolor fluorescence imaging with the machine learning technique was capable of providing comprehensive information of drought stress effect on the photosynthesis and the secondary metabolisms. The technique proposed in this study can be applied not only to the evaluation of drought stress in *Arabidopsis* but also to other crops.

## Author Contributions

HC and JY designed the experiment. JY, DS, HX, and HW performed the experiment. HC, JY, and DS contributed to the data analysis and wrote the manuscript. FY and YH provided suggestions on the experiment design and discussion sections. All authors read and approved the final manuscript.

## Conflict of Interest Statement

The authors declare that the research was conducted in the absence of any commercial or financial relationships that could be construed as a potential conflict of interest.

## Footnotes

1 http://www.scikit-learn.org/stable/index.html

## Abbreviations

PAM: pulse amplitude modulation
*Fv’/Fm’*: the trapping efficiency of photosystem-II
*Fs/Fo*: steady-state of chlorophyll fluorescence yield normalized to the minimal fluorescence yield
UV: ultraviolet
*osca1*: *Arabidopsis* of *reduced hyperosmolality-induced [Ca^2+^]_i_ increase 1*
MDA: malondialdehyde
CCD: charge-coupled device
LED: light-emitting diode
MF: measuring flash
AL 1: actinic light 1
SF: saturating flashes
AL 2: actinic light 2
*Fo*: the minimum fluorescence in the dark-adapted state
*Fm*: the maximum fluorescence in the dark-adapted state
*Fp*: the peak rise in fluorescence
*Ft_Ln*: the instantaneous fluorescence during light adaptation
*Ft_Lss*: the steady-state fluorescence in light
*Fm_Ln*: the maximum fluorescence during light adaptation
*Fm_Lss*: the steady-state maximum fluorescence in light
*Ft_Dn*: the instantaneous fluorescence signals before the saturating flashes
*Fm_Dn*: the maximum fluorescence signals during dark relaxation
*Fv/Fm*: the maximum quantum yield of PSII photochemistry
*NPQ_Ln*: instantaneous non-photochemical quenching during light adaptation
*NPQ_Lss*: steady-state non-photochemical quenching
*Rfd_Ln*: instantaneous fluorescence decline ratio in light
Φ*PSII_Lss*: steady-state PSII quantum yield
Φ*PSII_Ln*: PSII quantum yield during light adaptation
*qN_Dn*: coefficient of non-photochemical quenching during dark relaxation
*qN_Ln*: coefficient of non-photochemical quenching during light adaptation
*qL_Ln*: coefficient of photochemical quenching during light adaptation
*qL_Lss*: coefficient of photochemical quenching in steady-state
*Fv_Lss*: variable fluorescence in steady-state
*Fv_Ln*: variable fluorescence during light adaptation
*BF*: blue fluorescence
*GF*: green fluorescence
*RF*: red fluorescence
*IrF*: far-red fluorescence
TBA: thiobarbituric acid
TCA: trichloroacetic acid
ROIs: region of interests
ANOVA: analysis of variance
SFS: sequential forward selection
SVM: support vector machine
IrF/BF: fluorescence far-red/blue ratio
GF/BF: fluorescence green/blue ratio
BF/IrF: fluorescence blue/far-red ratio
ABA: abscisic acid
WT: wild-type
